# Bispecific antibody does not induce T-cell death mediated by chimeric antigen receptor against disialoganglioside GD2

**DOI:** 10.1080/2162402X.2017.1320625

**Published:** 2017-04-28

**Authors:** Sayed Shahabuddin Hoseini, Konstantin Dobrenkov, Dmitry Pankov, Xiaoliang L. Xu, Nai-Kong V. Cheung

**Affiliations:** a Department of Pediatrics, Memorial Sloan Kettering Cancer Center, New York, NY, USA; b Department of Surgery, Memorial Sloan Kettering Cancer Center, New York, NY, USA

**Keywords:** Bispecific antibody, chimeric antigen receptor, disialoganglioside, immunotherapy, T cell exhaustion

## Abstract

Chimeric antigen receptors (CAR) and bispecific antibodies (BsAb) are two powerful immunotherapy approaches for retargeting lymphocytes toward cancer cells. Despite their success in lymphoblastic leukemia, solid tumors have been more recalcitrant. Identifying therapeutic barriers facing CAR-modified (CART) or BsAb-redirected T (BsAb-T) cells should facilitate their clinical translation to solid tumors. Novel lentiviral vectors containing low-affinity or high-affinity 4-1BB second-generation anti-GD2 (disialoganglioside) CARs were built to achieve efficient T cell transduction. The humanized anti-GD2 × CD3 BsAb using the IgG-scFv platform was described previously. CART and BsAb-engaged T cells were tested for viability, proliferation, and activation/exhaustion marker expression, and *in vitro* cytotoxicity against GD2(+) tumor cells. The antitumor effect of CAR-grafted and BsAb-T cells was compared in a human melanoma xenograft model. The majority of high CAR density T cells were depleted upon exposure to GD2(+) target cells while the BsAb-T cells survived. The *in vitro* cytotoxicity of the surviving CART cells was inferior to that of the BsAb-T cells. Using low-affinity CARs, inclusion of the 4-1BB co-stimulatory domain or exclusion of a co-stimulatory domain, or blocking PD1 did not prevent CART cell depletion. Both CART cells and BsAb-T cells penetrated established subcutaneous human melanoma xenografts; while both induced tumor regression, BsAb was more efficient. The fate of T cells activated by BsAb differs substantially from that by CAR, translating into a more robust antitumor effect both *in vitro* and *in vivo*.

## Introduction

It is well known that T cells are effective for treating human cancers. Learning how to exploit these cells is essential. To mediate antitumor function, T cells need to infiltrate tumors, survive the tumor microenvironment, and perform their tumoricidal activity. Results of the previous studies have suggested that a combination of CD4^+^ and CD8^+^ T cells together should provide optimal antitumor effect,^1,2^ but both populations need to survive the microenvironment. Furthermore, inducing tumor cytotoxicity without excessive activation is critical to prevent activation-induced cell death (AICD). Currently, two approaches have been pursued to redirect polyclonal T cells: chimeric antigen receptors (CAR) and bispecific antibodies (BsAb). Their relative merit for each tumor target system is yet unclear.

Disialoganglioside (GD2) is an established target that is constitutively expressed in neuroblastoma and many other tumors.^3-5^ Antibody based immunotherapies directed at this target have proven efficacy.^6,7^ Cytotoxic effect of neuroblastoma immunotherapy is mostly mediated by NK cells and myeloid cells.^8^ T cells engineered against neuroblastoma have also shown promise.^9^ Since GD2 is present on multiple human cancers, novel GD2-directed immunotherapies can have broad clinical implications.

Similar to other solid tumor systems, CAR-retargeted T cells toward GD2 have met initial success,^10^ but T cell exhaustion and depletion upon antigen exposure soon became apparent. Various strategies have been developed to bypass AICD, including modifying the structure of CAR to avoid Fc receptor-mediated fratricide,^11,12^ incorporating 4-1BB instead of CD28 in the cytoplasmic domain of CAR,^13^ constitutive activation of akt/PI3K pathway,^14^ and antibody-mediated checkpoint blockade.^15^ Measuring both immune (i.e. T cell tumor infiltration and survival/persistence) and therapeutic (tumor regression) endpoints have been used for the assessment of antitumor effect. In our study, we directly compare functional efficacy of CAR versus BsAb in directing T cells to mediate antitumor effect and to study the role of CAR density versus CAR affinity in overcoming hurdles in implementing these T-cell based therapies.

## Results

### Anti-GD2 CART cells are depleted upon stimulation with GD2(+) target cells in vitro

To generate an anti-GD2 CART cell product, T cells generated from frozen PBMCs were transduced with the hu3F8CAR lentiviral vector. Human T cell transduction efficiency with hu3F8CAR was generally ≥80%.

**Figure 1.**
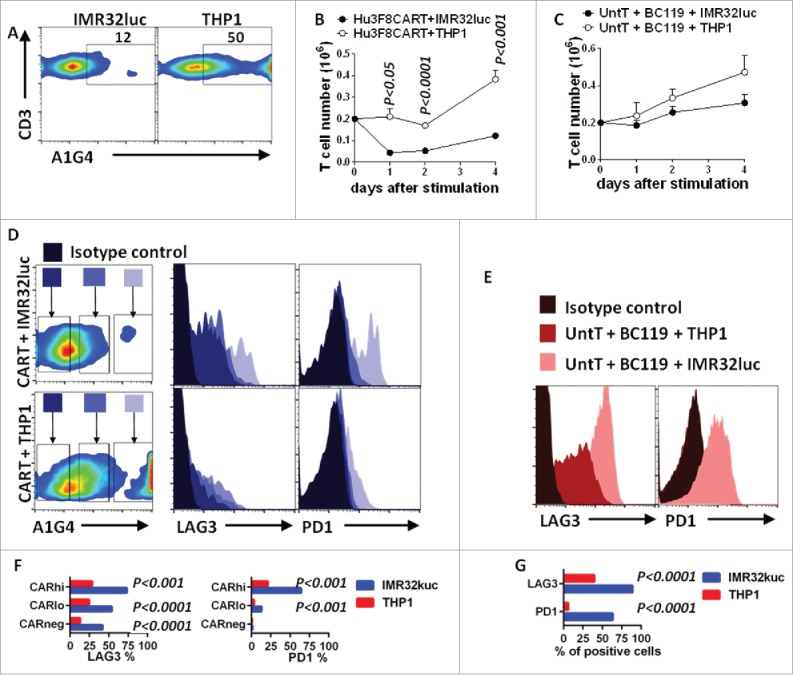
Anti-GD2 CART cells are depleted upon stimulation with GD2(+) target cells *in vitro*. (A, B) T cells that were transduced with the hu3F8CAR (anti-GD2) and expanded for 8 d thereafter were stimulated with irradiated IMR32luc (GD2+) or THP1 (GD2−) cells. After 1, 2, and 4 d, cells were counted and CAR expression was assessed by anti-idiotype antibody (A1G4). Dead cells were excluded by Annexin-V staining. Absolute cell number was calculated based on cell count and flow cytometry. (C) T cells that were cultured similar to CART cells but were not transduced (UntT) were stimulated with irradiated IMR32luc (GD2+) or THP1 (GD2−) cells in the presence of BC119 (0.01 μg/mL). After 1, 2, and 4 d, cells were counted and assessed by flow cytometry. Absolute cell number was calculated as explained for CART cells. (D–G) One day after stimulation, cells were assessed by flow cytometry. The anti-idiotype A1G4 antibody was used to stain CART cells. CAR^hi^, CAR^lo^, and CAR^neg^ cells were then gated for the expression of PD1 and LAG3. FACS plots are gated on human CD3(+) T cells. Data were generated from triplicates. Student *t* test was used for statistical analysis.

To assess the GD2 specificity of the CART cells, T cell activation was studied using irradiated GD2(+) IMR32luc and GD2(−) THP1 targets. CART cells produced both T helper-1 (TH1) cytokines (interferon-γ (IFNγ), tumor necrosis factor-α (TNFα), and interleukin-2 (IL2)) and TH2 cytokines (IL4 and IL6) in an antigen-specific manner (Fig. S1). Unexpectedly, analysis of T cell viability within 24 h showed that the majority of cells expressing high levels of hu3F8CAR (CAR^hi^) were depleted when exposed to GD2(+) IMR32luc target cells but remained intact when GD2(−)THP1 targets were used (Fig. 1A and B). In contrast, in the presence of BC119 BsAb, exposure of untransduced T cells to GD2(+) or GD2(−) target cells did not compromise survival or cell proliferation (Fig. 1**C**). Antigen-specific loss of CART cells followed the same dynamics when exposed to solid-phase bound purified GD2 or anti-3F8-idiotype antibody A1G4^16^ whereas those exposed to solid-phase-bound control ganglioside GD3 or control anti-5F11-anti-idiotype antibody 1G8 survived (Fig. S2). In addition, analysis of the surviving cells revealed that while PD1 and LAG3 were significantly upregulated on CAR^hi^ cells when stimulated with IMR32luc, their expression following stimulation with GD2(−) targets were modest (Fig. 1D and F). Similarly, BsAb-stimulated T cells upregulated their PD1 and LAG3 expression in an antigen-specific manner (Fig. 1E and G). However, this PD1 overexpression on BC119-stimulated T cells did not compromise cell survival suggesting that these markers might be activation rather than exhaustion markers in these experiments. Analysis of PD1 and LAG3 expression over time showed that these markers were upregulated as early as 5 h after stimulation with target cells (Fig. S3). Furthermore, incubation of CART cells with a PD1-blocking antibody pembrolizumab did not prevent cell loss upon exposure to IMR32luc targets (data not shown).

### Low-affinity anti-GD2 CARs cannot prevent CART cell depletion upon antigen exposure

Humanized 3F8 (hu3F8) and 5F11(F104) were both anti-GD2 antibodies that were affinity-matured to generate hu3F8(D32H-E1K) and 5F11(Y104) species, respectively.^17-19^ The affinity (*K_D_*) of the scFv format of hu3F8, hu3F8(D32H-E1K), 5F11(F104), and 5F11(Y104) is 153.4 nM, 3.6 nM, 19.8 nM, and 6.02 nM, respectively.^18,19^

To investigate whether CARs with lower affinities can prevent CART cell overstimulation and subsequent death, the scFv of hu3F8 and hu3F8(D32H-E1K) or 5F11(F104) and 5F11(Y104) were used for generation of CARs with different affinities.

**Figure 2.**
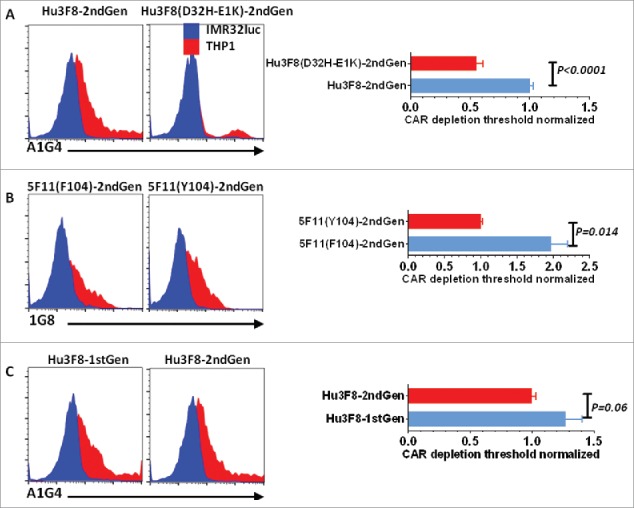
Lowering CAR affinity cannot prevent CART cell depletion upon antigen exposure. T cells that were transduced with the (A) 2nd-generation hu3F8 (lower-affinity) or hu3F8(D32H-E1K) (higher-affinity) CARs, (B) 2nd-generation 5F11(F104) (lower-affinity) or 5F11(Y104) (higher-affinity) CARs, and **(C)** 1st-generation or 2nd-generation hu3F8 CARs and expanded thereafter for 8 d were stimulated with irradiated IMR32luc (GD2+) or THP1 (GD2-) cells. One day later, cells were assessed by flow cytometry for CAR expression. Anti-idiotype antibodies (A1G4 for hu3F8CAR or hu3F8(D32H-E1K) and 1G8 for 5F11(F104) or 5F11(Y104)CAR) were used for CAR staining. Dead cells were excluded by Annexin-V staining. CART cells stimulated for 1 d with targets cells (as mentioned above) and Quantum beads were stained with anti-idiotype antibodies and assessed by flow cytometry. Mean fluorescence intensity (MFI) of stained Quantum beads was used for generation of a standard curve using a calculator provided by the manufacturer. The average number of CAR molecules expressed per cell was interpolated using the CAR MFI and the standard curve. A depletion threshold was defined as the CAR density that separated top 5% from the bottom 95% in the surviving population. Date were normalized based on 2nd-generation hu3F8 or 5F11(Y104) thresholds. Pooled results of two independent experiments in triplicates are shown.

**Figure 3.**
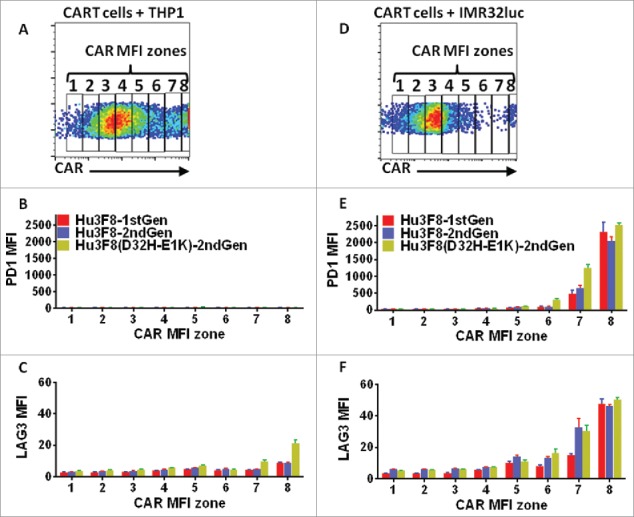
Higher avidity CARs predispose T cells to exhaustion more than lower avidity CARs do. CART cells were generated as explained in Fig. 2. One day after stimulation with target cells in triplicates, cells were assessed by flow cytometry. (A, D) According to the mean fluorescent intensity (MFI), the level of CAR expression was divided into eight zones. Expression of the exhaustion markers PD1 (B, E) and LAG3 (C, F) was assessed on CD3(+) CART cells in each zone using flow cytometry.

Following exposure to GD2(+) targets, a near complete CAR^hi^ cell depletion for all four CART cell types were observed (Fig. 2A and B). Since CART cells with higher CAR expression were more susceptible to depletion, we quantified the average minimal number of CAR molecules on a given T cell population that predisposed them to depletion upon antigen encounter. The minimum detection threshold by Quantum bead quantification was around 4,000 molecules per cell. The maximum CAR expression exceeded 2,000,000 molecules per cell. A depletion threshold was defined as the CAR density that separated top 5% from the bottom 95% in the surviving population. The average depletion threshold for the two parental clones of hu3F8- and 5F11(F104)-CART cells was higher than the threshold for the affinity matured clones of hu3F8(D32H-E1K)- and 5F11(Y104)-CART cells (Fig. 2A and B). While lower affinity CART cells were statistically less prone to depletion, almost all of the anti-GD2 CAR^hi^ T cells, regardless of their affinity, were depleted upon exposure to GD2(+) targets (Fig. 2A and B). To further investigate the effect of CAR affinity on T cell exhaustion, expression of PD1 on hu3F8 and hu3F8(D32H-E1K) CART cells was assessed after target exposure. PD1 level was positively correlated with CAR expression on the surviving T cells stimulated with GD2(+) tumor cells. Importantly, high-affinity hu3F8(D32H-E1K) CART cells expressed more PD1 than low affinity hu3F8CART cells consistent with an association of T cell activation/exhaustion phenotype with high affinity CARs (Fig. 3A, B and D, E). LAG3 expression on CART cells exposed to GD2(+) targets was also increased though less pronounced than that of PD1 (Fig. 3C and F). Because of the lack of a co-stimulatory domain, 1st-generation CARs should provide weaker signaling than 2nd-generation CARs where the 4-1BB domain provides a survival signal to the CART cells. Stimulation with GD2(+) target cells caused similar depletion of the 1st- and 2nd-generation hu3F8CART cells (Fig. 2C) that was accompanied by upregulation of PD1 and LAG3 (Fig. 3).

### GD2-specific CART cell depletion severely compromises CART cell-mediated cytotoxicity in short-term killing assays

**Figure 4.**
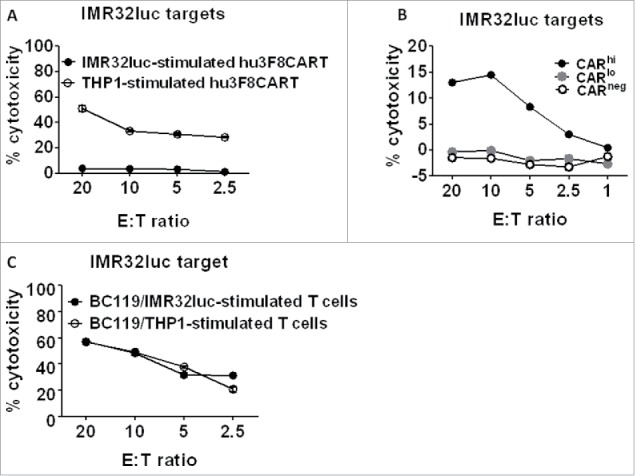
GD2-specific CART cell depletion severely compromises their function in short-term killing assays. (A) T cells that were transduced with the hu3F8CAR and expanded thereafter for 8 d were stimulated with irradiated IMR32luc (GD2+) or THP1 (GD2−) cells. Four days later, hu3F8CART cells were tested against IMR32luc targets in a 4-h ^51^Cr-release assay. (B) T cells that were transduced with the hu3F8CAR and expanded thereafter for 8 d were sorted for the CAR^hi^, CAR^lo^, and CAR^neg^ populations and were tested in a 4-h ^51^Cr-release assay. (C) Untransduced T cells were similarly stimulated with irradiated target cells but in the presence of BC119 (0.01 μg/mL). Four days later, BC119-activated T cells were tested against IMR32luc targets in a 4-h ^51^Cr-release assay in the presence of BC119 (0.01 μg/mL).

Following antigen exposure for 4 d with either irradiated IMR32luc or THP1 cells, hu3F8CART cells or BC119-redirected T cells were tested against IMR32luc targets in a 4-h ^51^Cr-release assay. Whereas THP1-exposed CART cells lysed target cells efficiently, IMR32luc-exposed CART cells lost their cytotoxic function almost completely (Fig. 4A). To confirm the compromised cytotoxicity of the surviving CAR^lo^ cells, CART cells were sorted for CAR^hi^, CAR^lo^, and untransduced (CAR^neg^) cells and their cytotoxicity against IMR32luc was determined in a 4-h chromium release assay. While CAR^hi^ cells demonstrated cytotoxicity, CAR^lo^ cells had compromised killing of target cells (Fig. 4B). In contrast to CART cells, exposure of T cells to BC119 in the presence of either irradiated GD2(+) IMR32luc or GD2(−) THP1 cells did not affect their cytotoxicity (Fig. 4C).

### Surviving CART cells retain significant antitumor cytotoxicity in long-term killing assays

**Figure 5.**
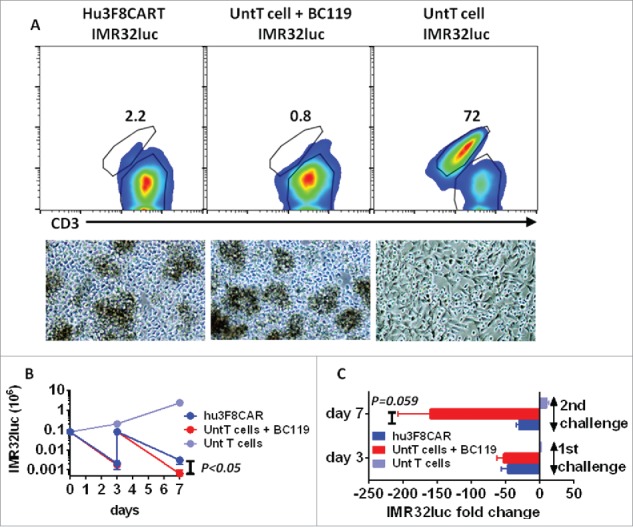
Surviving CART cells retain a significant antitumor cytotoxicity in long-term killing assays. (A) Hu3F8CART cells or untransduced T cells (+/− 0.01 μg/mL of BC119) were exposed to GD2(+) IMR32luc cells in triplicates for 3 d at an E:T = 1. On day 3, cells were harvested, counted, and mixed with fresh IMR32luc targets (E:T = 1). After 4 more days, cells were counted and assessed by flow cytometry. Dead cells were excluded by Annexin V and CD3 staining was used to differentiate between CD3(+) T cells and CD3(−) targets. Photo micrographs of cultures at day 7 are shown. (B, C) Number of live target cells was calculated based on cell count and flow cytometry.

Depletion of CAR^hi^ T cells severely affected antitumor cytotoxicity in a short-term ^51^Cr-release assay. The antitumor potential of the remaining CAR with low density (CAR^lo^) cells was tested in a long-term killing assay. Hu3F8CART cells or untransduced T cells (+/− of BC119) were exposed to GD2(+) IMR32luc cells for 3 d at a low effector:target ratio (E:T = 1). On day 3, cells were harvested, counted, and mixed with fresh IMR32luc targets at the same E:T ratio. After 4 days, cells were counted and assessed by flow cytometry. Annexin V was used to exclude dead cells and staining for CD3 was used to differentiate between CD3(+) T cells and CD3(−) targets. Target cell killing over the first 3 d reflected the combined antitumor cytotoxicity by CAR^hi^ cells and the slow antitumor cytotoxicity by the surviving CAR^lo^ cells. After antigen induced depletion of CAR^hi^ cells on day 1, the surviving CART cells harvested after 3 d were CAR^lo^. The long-term killing effect (from day 3 to 7) of CAR^lo^ cells was then quantified. As shown in Fig. 5, even though CAR^hi^ T cells died after antigen exposure, the tumor cells in contact with CAR^hi^ T cells were also eliminated, a fate similarly shared by target cells exposed to BC119-redirected T cells. From day 3 through day 7, the surviving CAR^lo^ T cells were able to kill target cells although at significantly lower efficiency than BC119-activated T cells. In the control groups, target cells exposed to untransduced T cells in the absence of BC119 grew rapidly and overtook the T cells. These data confirmed the residual functionality of CAR^lo^ cells that could be underestimated in a 4 h ^51^Cr-release assay.

### Anti-GD2 bispecific antibody-redirected T cells outperform CART cells against tumor cells in vivo

**Figure 6.**
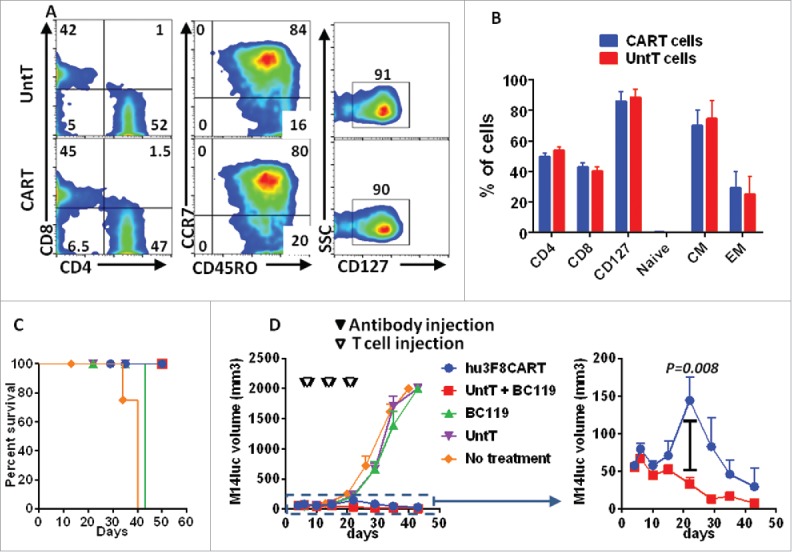
Anti-GD2 BsAb-redirected T cells cure melanoma tumors with a faster kinetics than CART cells *in vivo*. (A, B) T cells were stimulated with CD3/CD28 beads for 3 d and then transduced with hu3F8CAR or left untransduced. Four days after transduction and just before adoptive transfer, cells were phenotyped by flow cytometry. (C, D) Immunodeficient DKO mice were subcutaneously inoculated with GD2(+) M14luc human melanoma xenografts (4 × 10^6^ cells). Therapy was started after 7 d. Mice were divided into five groups: (1) recipients of hu3F8CART cells (10 million/weekly for 3 weeks), (2) recipients of untransduced T cells (10 million/weekly for 3 weeks) plus anti-GD2 × anti-CD3 BsAb (BC119, 50 μg/twice weekly for 3 weeks), (3) recipients of BC119 only (50 μg/twice weekly for 3 weeks), (4) recipients of T cells only (10 million/weekly for 3 weeks), (5) recipients of no therapy. All mice including those with no therapy received subcutaneous injection of interleukin-2 (1000 IU/ 2–3 times per week). Tumor volume was measured weekly. For survival analysis, either death or tumor ≥2000 mm^3^ was scored as event in the survival analysis.

To compare the functionality of hu3F8CART cells versus T cells redirected by BC119 against tumor cells *in vivo*, a GD2(+) melanoma cell line M14luc was xenografted subcutaneously in immunodeficient DKO mice. Hu3F8CART cells or untransduced T cells that were cultured for 7 d *in vitro* were used for *in vivo* experiments. Transduction efficiency of CAR T cells for *in vivo* experiments determined on day 7 post-transduction was confirmed to be more than 80%. Subpopulation analysis showed that the percentage of CD4^+^ T cells was slightly higher than CD8^+^ T cells. Most of the cells expressed surface markers of central memory cells (80% by FACS) (Fig. 6A and B). T cells were injected intravenously on day 7, 14, and 21 after tumor inoculation. BC119 was injected one day before and one day after each T cell injection. To support T cell survival *in vivo*, low dose IL-2 was injected to all mice including the controls. Mice receiving either BC119 alone or untransduced T cells alone were used as controls. Neither BC119 nor untransduced T cells by itself conferred any antitumor effect, whereas the combination of the two provided rapid shrinkage of the established tumors. In contrast, tumor mass in the CART cell group, despite initial fast growth when compared with the BC119+T cell group, also showed regression with longer follow-up (Fig. 6C and D). These results are consistent with the *in vitro* observations of the two phases of cytotoxicity, short-term and long-term, confirming a small advantage of BC119-redirected T cells over hu3F8CART cells in this particular melanoma model.

### The fate of CART cells and BsAb-engaged T cells differs at the tumor site in vivo

**Figure 7.**
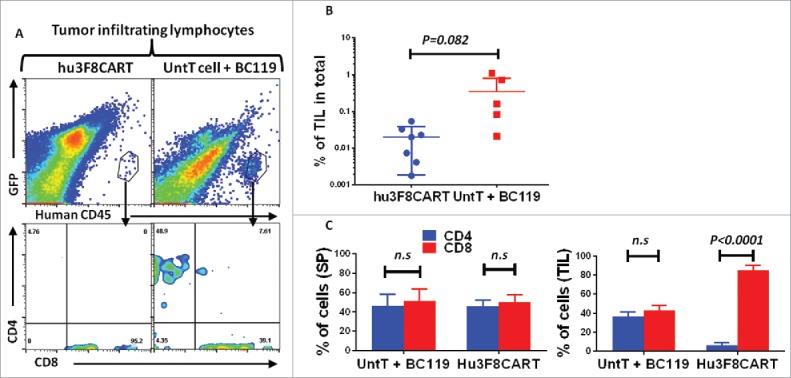
The fate of CART cells and BsAb-engaged T cells differs at the tumor site *in vivo*. (A–C) Mice treated similarly as in Fig. 5 but in the absence of *in vivo* IL2 injection were killed one day before and 2 d after the third T cell injection. Splenocytes and tumor infiltrating lymphocytes (TILs) were assessed by flow cytometry the same day. Data were pooled (*n* = 7 and *n* = 6 for the CART cell and untransduced T cells plus BC119 (UntT cell + BC119) groups, respectively). Human CD45(+) cells were gated for analysis.

Infiltration of T cells into solid tumor masses is a prerequisite for a robust therapeutic response. To study if hu3F8CART cells and BC119-redirected T cells could penetrate tumors, mice were killed on day 22 and 25 of the experiment. Splenocytes and tumor infiltrating lymphocytes (TILs) were assessed by flow cytometry. The percentage of TILs in the BC119-engaged T cell group was significantly higher than that in the CART cell group (Fig. 7A and B). The ratio of CD4^+^ and CD8^+^ T cells in the spleen of mice in BsAb-engaged versus CART cell groups was comparable. Surprisingly, whereas the percentage of CD4^+^ and CD8^+^ TILs was nearly equal in the BC119-T cell group, almost all of the hu3F8CART cells were expressing CD8^+^ (Fig. 7A and C). Numerical advantage of TILs having a balanced CD4^+^ and CD8^+^ T cell phenotype in the BC119-redirected versus CART cell group could partly explain the superior antitumor effect mediated by BsAb versus CAR in these *in vivo* experiments.

## Discussion

By directly comparing CAR versus BsAb in redirecting T cells toward GD2, we showed that CAR was associated with substantial T cell death, resulting in lower antitumor potency. This depletion was antigen-specific, induced within 24 h after exposure to solid phase antigen, cell bound antigen, or anti-idiotype antibodies, not preventable by 4-1BB-signaling, or by anti-PD1 checkpoint blockade. Furthermore, exhaustion and depletion was preferential for T cells with high CAR density and was unaffected by lowering scFv affinity. *In vivo*, the input CD4^+^ versus CD8^+^ ratio was maintained when TILs were driven by BsAb, and substantially reduced when driven by CAR. BsAb driven T cells demonstrated a more robust antitumor effect *in vivo* without evidence of increased toxicity.

The phenomenon of AICD for T cells is well known. With GD2 CART cells the evidence is unequivocal. The immunology behind AICD is vital to the central property of the immune system to put brakes on run-away immune cells to prevent autoimmunity. The signaling pathways for AICD of T cells have been well defined. For CART cells they include phosphorylation of ERK, AKT, and Stat6.^15^ Various strategies have been developed to bypass CART cell AICD, such as modifying CAR structure,^11-13^ constitutive activation of survival pathways,^14^ and using immune checkpoint inhibitors.^15^ In our CAR design, we avoided the CH2-CH3 FcR binding domain, incorporated 4-1BB instead of CD28, and applied anti-PD1 antibodies. Yet, none of these methods was able to alleviate AICD of CART cells. Instead, we directed our efforts to determine the role of CAR density and affinity to study the following endpoints: T cell tumor infiltration, T cell phenotype inside the tumor, and antitumor effect *in vitro* and against tumor xenografts in mice. Both density and affinity could enhance T cell activation and hence AICD. Our findings were unexpected. While high density CART cells died, low density CART cells persisted in the presence of GD2(+) tumors and were able to mediate an effective although delayed antitumor effect. One implication of these findings is the identification of a CAR density threshold which could serve as the receptor ceiling for CART cell therapy in the clinic. The other surprising result was the inability to avoid T cell exhaustion and T cell death despite lowering the receptor affinity for the tumor antigen. It was reported that 4-1BB signaling reduces anti-CD19 CART cell exhaustion;^13^ however, we observed similar GD2-specific depletion of T cells bearing a 1st-generation (lacking 4-1BB) or 4-1BB -containing 2nd-generation CAR. Collectively, these data suggest that GD2 CART cell depletion was more dependent on the density of CAR on the cell surface than on the affinity of the CAR itself. In fact, it was recently reported that acquisition of an exhausted phenotype by T cells was determined more by the frequency of T cell receptor (TCR) engagement than with the strength of TCR engagement.^20^ Recently, Tanaka et al. showed that varicella zoster virus (VZV)-specific T-cells expressing a CAR for GD2 lose their GD2 specificity after encountering GD2(+) target cells while they retain specificity toward VZV.^21^ Our data suggest that this loss of function could be due to depletion of CART cells leaving the rest of cells (which are VZV specific) functional against VZV.

T cell survival at the site of tumor is generally accepted as a critical first step to achieve a robust clinical response. The persistence of even low levels of circulating CART cells beyond 6 weeks was reported to bestow a progression-free survival advantage.^10^ Our data showed that the percentage of TILs redirected via BsAb was higher compared with those engaged by CAR. This could be due to superior proliferation mediated by the BsAb than by the CAR. Additionally, the depletion phenomenon that happened *in vitro* could also paralyze CART cell at the site of tumor *in vivo*. It is possible that the AICD phenomenon observed in the GD2 system was antigen-dependent and/or tumor dependent since a recent study on mesothelin-specific CART cells did not find depletion of T cells at the tumor site compared with cells homing to the spleen.^22^

To maintain comparability, we kept near identical T cell subset ratios and central memory characteristics for CART versus BsAb-driven T cells at the time of adoptive transfer. We also used a relatively short duration for *in vitro* culture before *in vivo* administration to ensure that the T cells were not terminally differentiated. In our experiments, we observed a balanced CD4^+^and CD8^+^ TIL phenotype in the BsAb-treated mice; however, CD4^+^ TILs were almost absent in the CART cell-treated mice. Since CD4^+^ T cells play important roles in antitumor response (both direct cytotoxicity and providing help for CD8^+^ T cells^23,24^), their absence among CAR TILs could compromise antitumor response that is CD4^+^ T cell dependent.

Our data support an advantage of BsAb over CAR in redirecting T cells for tumor therapy. Although there was no obvious difference in their toxicity profiles in these preclinical models, a direct comparison in patients will be necessary before the final verdict on their clinical utility can be decided. While both modalities use polyclonal T cells, CART cell technology is patient-specific where each injection requires custom synthesis, distinct from BsAb which is manufactured and distributed like any other pharmaceutical drug. Unlike liquid tumors, TILs have to survive in the tumor microenvironment and if they cannot persist, single CART injections will unlikely have long-term benefit. Multiple CART cell injections would substantially increase the cost of treatment, defeating its original premise that genetically modified killer cells, unlike BsAb, should persist for years. BsAbs have been through or undergoing phase I clinical trials. The anti-CD19 BsAb format called BiTE® (Blinatumomab) was recently FDA approved. Main toxicities have been cytokine release syndrome, as was observed for CART cells.^25^ The GD2-BsAb used in these studies was built with a silent Fc to reduce or to prevent AICD and cytokine storm. If clinically proven to be safe, it could be an alternative or replacement of CART cells.

## Materials and methods

### CAR design and vectors

Humanized 3F8 (hu3F8) and 5F11(F104) are two anti-GD2 antibodies that were affinity-matured to generate hu3F8(D32H-E1K) and 5F11(Y104) species, respectively.^17-19^ The anti-GD2 scFv encoding sequences were cloned into an anti-CD19 CAR construct in place of the anti-CD19 scFv. The original CD19 CAR contained the human CD8^+^ leader, CD8^+^ hinge-transmembrane domain, the human 4-1BB molecule, and the CD3 ζ endodomain, as described previously.^26^ The hu3F8, hu3F8(D32H-E1K), 5F11(F104), or 5F11(Y104) CAR cassettes were inserted into BE plasmid^27^ using In-Fusion cloning method (Clontech, Mountain View, CA). To generate a 1st-generation hu3F8 CAR, the 4-1BB domain was removed using PCR. The rest of the CARs are 2nd-generation. All experiments were performed with 2nd-generation CARs unless otherwise specified.

### Lentivirus production

Lentiviruses were produced by transfection of 2 × 10^7^ 293T cells using 20 μg lentiviral vector, 10 μg pVSV-G, 20 μg pCMV-dR8.91,^28^ and 100 μL Polyjet (SignaGen, Rockville, MD) in 175 cm^2^ tissue culture flasks. Briefly, plasmid DNA and Polyjet reagent were mixed with 1.5 mL DMEM medium in two separate 50 mL conical tubes. After 5 min, Polyjet solution was added to the DNA mixture and left at room temperature for 20 min. DNA-Polyjet complex was then admixed with 2 × 10^7^ 293T cells resuspended in 1.5 mL DMEM supplemented with 10% fetal bovine serum (FBS, Life Technologies), penicillin/streptomycin, and glutamate. The resulting mixture was incubated at 37°C in a rotator for 30 min before transferred to flasks with 15 mL DMEM culture medium. Medium was exchanged in 24 h. Virus harvested 48 and 72 h after transfection was combined, centrifuged at 25,000 rpm (100,000 G) for 2 h, and resuspended in 1 mL RPMI culture medium.

### T cell transduction

T cells were purified from human PBMCs using a Pan T-cell isolation kit (Miltenyi Biotec, Auburn, CA), and then activated and expanded with CD3/CD28 Dynabeads (Invitrogen, Carlsbad, CA) according to the manufacturer's protocol.RPMI supplemented with 10% heat-inactivated FBS and 30 IU/mL interleukin-2 (IL-2) was used for T cell culture. Three days after activation, 1–4 × 10^6^ of human T cells were transduced with 1 mL of concentrated virus supplemented with protamine sulfate (4μg/mL) and IL-2 (30 IU/mL) followed by rotator shaking for 1 h at 37°C. The concentrated virus was generated by centrifugation of 35 mL of viral supernatant at 100,000 G speed for 2 h. Cells were then seeded into retronectin (Takara Bio USA)-coated (50 ug/mL, 250 uL/well) 24-well plates (1 million/well in 2 mL total volume), centrifuged at 800 g/32°C /90 min, and transferred to incubator at 37°C with 5% CO_2_. After 14–16 h, fresh media and IL-2 were added to the cells. For experiments with BsAb, T cells were similarly treated but not transduced with the virus. T cells were split every 2 d for 4–5 d in IL-2 containing media. Then, T cells were harvested, washed, and resuspended in PBS for injection.

### Bispecific antibodies (BsAb)

The hu3F8 BsAb was designed using VH/VL domains from hu3F8 antibody and huOKT3 scFv fused to the C-terminus of the light chain of a human IgG1 as described previously.^17,29,30^ The hu3F8 × huOKT3 BsAb was named biclone-119 (BC119).

### Cytotoxicity assay (chromium-51 release assay)

GD2-expressing (IMR32luc, obtained from American Type Culture Collection (ATCC), Manassas, VA) and GD2-negative leukemia cells (THP1) were cultured in RPMI1640 (Cellgro) supplemented with 10% fetal bovine serum (FBS, Life Technologies) at 37°C in a 5% CO_2_ humidified incubator. Adherent cells were harvested with EDTA/trypsin. T cells were purified from human PBMC using Pan T cell isolation kit (Miltenyi Biotec). Target tumor cells were labeled with sodium 51Cr chromate (Amersham, Arlington Height, IL) at 100 μCi/10^6^ cells at 37°C for 1 h. After the cells were washed twice, 5,000 target cells/well were mixed with 50,000 effector cells (hu3F8 CAR or non-modified T) cells at E:T = 10:1 in the presence of serial dilutions of BsAb antibodies, and incubated in 96-well polystyrene round-bottom plates (BD Biosciences) in a final volume of 250 μL/well, at 37°C for 4 h. After centrifugation at 400 g for 10 min, the released ^51^Cr in the supernatant was counted in a γ-counter (Packed Instrument, Downers Grove, IL). Percentage of specific lysis was calculated using the formula 100% (experimental cpm − background cpm)/(total cpm − background cpm), where cpm represented counts per minute of ^51^Cr released. Total release was assessed by lysis with 10% SDS (Sigma, St Louis, Mo), and background release was measured in the absence of effector cells.

### Cytokine release assay

The hu3F8 CART cells or unmodified T cells in the presence of hu3F8-BsAb (80,000/per well) were co-cultured with irradiated IMR32luc or THP1 cell lines (80,000/ per well) in 37°C in 24 well plate. Supernatants were harvested after 24 h of culture. Concentration of cytokines were assessed using an ELISA based cytokine assay kit (OptEIA™ human cytokine set, BD Biosciences, San Jose, CA) according to the manufacturer's instructions.

### FACS analysis

Hu3F8/hu3F8(D32H-E1K) and 5F11(F104)/5F11(Y104) CAR expression was detected with rat anti-idiotypic antibodies A1G4 and 1G8 (conjugated to Alexa Fluor 647 (Thermo Fisher Scientific)), respectively.^16^ Antihuman antibodies against CD3, CD4^+^, CD8^+^, CD45RO, CCR7, LAG3, PD1, and CD127 (BD Biosciences) were used to define T cell subpopulations. Stained cells were processed with a FACScalibur instrument (BD Biosciences) and analyzed with FlowJo software (FlowJo, LLC, Ashland, OR). The number of CAR molecule expression on T cells was determined using Quantum™ Simply Cellular® anti-Rat IgG (Bangs Laboratories, Inc.) microbeads according to the manufacturer's instructions.

### In vivo studies

All mouse experiments were performed in compliance with the Institutional Animal Care and Use Committee guidelines. The immunodeficient mice BALB-*Rag2*^−/−^IL-2R-γc-KO (DKO) were either purchased from Taconic Inc. (Hudson, NY) or maintained at MSK and provided with Amoxicillin food. For tumor challenge experiments, 6- to 8-week-old male mice were inoculated subcutaneously with 4 × 10^6^ GD2(+) M14luc melanoma cell line in Matrigel (BD Biosciences). Activated and transduced hu3F8CAR or untransduced T cells that were in culture for 7 d were intravenously injected weekly for 3 weeks starting 7 d after tumor inoculation. To support T cell survival *in vivo*, 1000 IU of interleukin-2 (IL2) was subcutaneously injected 3 times per week unless otherwise specified. Tumor size was measured weekly using Peira TM900 imaging device (Peira, Belgium).

## Supplementary Material

KONI_A_1320625_Supplementary_Figures.docx
