# Supplementary material for: Bispecific antibody does not induce T-cell death mediated by chimeric antigen receptor against disialoganglioside GD2
Source: Oncoimmunology. 2017 Apr 28;6(6):e1320625. doi: 10.1080/2162402X.2017.1320625 (PMC5486173; doi:10.1080/2162402X.2017.1320625)
Supplement: KONI_A_1320625_Supplementary_Figures.docx [file koni-06-06-1320625-s001.docx]

**Supplementary materials**

**Fig S1.** Anti-GD2 CART cells and BsAb-redirected T cells generate proinflammatory cytokines upon stimulation with GD2(+) target cells. T cells that were transduced with the hu3F8CAR or left untransduced were expanded for 8 days and then were stimulated with irradiated IMR32luc (GD2+) or THP1 (GD2-) cells. Untransduced T cells received anti-GD2×anti-CD3 BsAb (BC119, 0.01μg/ml) in the culture media. Supernatant was harvested after 24 hours and assessed for interferon-γ (IFNγ), tumor necrosis factor-α (TNFα), interleukin-2 (IL2), IL4, and Il6 using ELISA assay.


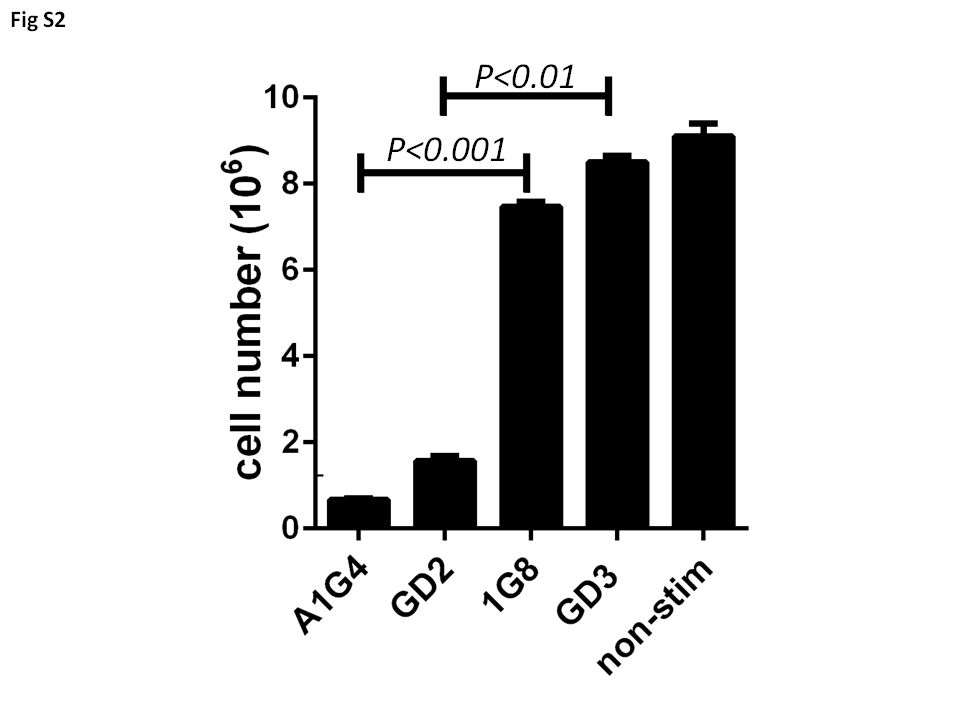


**Fig S2.** Anti-GD2 CART cells are depleted upon stimulation with surface-bound stimulatory antigens *in vitro*. Hu3F8CART cells (2 × 10^6^ cells/well) were co-incubated with surface-bound antigens. For that, GD2 or GD3 antigens (final concentration 1 μg/ml) or anti-3F8-idiotype antibody A1G4 or control anti-5F11-idiotype antibody 1G8 (final concentration 10 μg/ml) were coated overnight in 6-well non-culture plates at 4C. Cell count and viability was determined to investigate CART cell depletion.

**Fig S3.** PD1 and LAG3 are overexpressed on CAR and BsAb redirected T cells upon antigen stimulation. T cells that were transduced with the hu3F8CAR (anti-GD2) or untransduced T cells were expanded for an additional 8 days and were stimulated with irradiated IMR32luc (GD2+) cells. Untransduced T cells received BC119 (0.01μg/ml) or humanized anti-CD3 antibody OKT3 (30μg/ml) as a positive control for T cell activation. Expression of PD1 and LAG3 was determined on the cells via flow cytometry just before and also after 1.5, 5, and 24 hours of stimulation.
